# Ryanodine receptor modulation by caffeine challenge modifies Na^+^ current properties in intact murine skeletal muscle fibres

**DOI:** 10.1038/s41598-020-59196-9

**Published:** 2020-02-10

**Authors:** Sahib S. Sarbjit-Singh, Hugh R. Matthews, Christopher L.-H. Huang

**Affiliations:** 1 Physiological Laboratory, University of Cambridge, Downing Street, Cambridge, CB2 3EG United Kingdom; 2 Department of Biochemistry, University of Cambridge, Tennis Court Road, Cambridge, CB2 1QW United Kingdom

**Keywords:** Physiology, Neurology

## Abstract

We investigated effects of the ryanodine receptor (RyR) modulator caffeine on Na^+^ current (*I*_Na_) activation and inactivation in intact loose-patch clamped murine skeletal muscle fibres subject to a double pulse procedure. *I*_Na_ activation was examined using 10-ms depolarising, *V*_1_, steps to varying voltages 0–80 mV positive to resting membrane potential. The dependence of the subsequent, *I*_Na_ inactivation on *V*_1_ was examined by superimposed, *V*_2_, steps to a fixed depolarising voltage. Current-voltage activation and inactivation curves indicated that adding 0.5 and 2 mM caffeine prior to establishing the patch seal respectively produced decreased (within 1 min) and increased (after ~2 min) peak *I*_Na_ followed by its recovery to pretreatment levels (after ~40 and ~30 min respectively). These changes accompanied negative shifts in the voltage dependence of *I*_Na_ inactivation (within 10 min) and subsequent superimposed positive activation and inactivation shifts, following 0.5 mM caffeine challenge. In contrast, 2 mM caffeine elicited delayed negative shifts in both activation and inactivation. These effects were abrogated if caffeine was added *after* establishing the patch seal or with RyR block by 10 μM dantrolene. These effects precisely paralleled previous reports of persistently (~10 min) increased cytosolic [Ca^2+^] with 0.5 mM, and an early peak rapidly succeeded by persistently reduced [Ca^2+^] likely reflecting gradual RyR inactivation with ≥1.0 mM caffeine. The latter findings suggested inhibitory effects of even resting cytosolic [Ca^2+^] on *I*_Na_. They suggest potentially physiologically significant negative feedback regulation of RyR activity on Na_v_1.4 properties through increased or decreased local cytosolic [Ca^2+^], Ca^2+^-calmodulin and FKBP12.

## Introduction

Skeletal muscle excitation-contraction coupling involves triggering of a Na_v_1.4 mediated action potential. This is detected by voltage-dependent transverse tubular Ca_v_1.1 that undergoes transitions resulting in an allosterically-activated ryanodine receptor (RyR) mediated release of sarcoplasmic reticular (SR) Ca^2+^ thereby increasing cytosolic free Ca^2+^ concentration, [Ca^2+^]_i_, Recent findings have been suggestive of feedback mechanisms through which downstream events in this feedforward sequence might conversely affect Na_v_1.4 function. However, many of these reports provided structural rather than functional evidence, or involved experiments in cell lines or isolated cultured cells. They thus provide necessary rather than sufficient evidence for the actual operation of Ca^2+^ mediated feedback mechanisms in working muscle *in vivo*. Few available reports examined the existence and properties of RyR-mediated feedback actions on Na_v_1.4 function in intact *in situ* skeletal myocytes particularly those directly targeting the RyR mediated Ca^2+^ release process.

First, Na_v_1.4 possesses several potential Ca^2+^ and calmodulin (CaM) binding or modulation sites. These include: (a) one or more Ca^2+^-binding C-terminal EF-like hand motifs^1^; (b) a C-terminal isoleucine-glutamine (IQ) domain that could bind CaM following combination of Ca^2+^ with CaM’s own EF-hand motifs^2^; (c) a III-IV loop Ca^2+^/CaM-binding site^3^; (d) sites phosphorylatable by CaM kinase II (CaMKII) themselves regulated by Ca^2+^/CaM^4^ and (e) a phosphorylation site for phosphokinase C (PKC)^5^.

Secondly, single-cell patch-clamp studies reported that both rapid photo-release of caged Ca^2+^, raising [Ca^2+^]_i_ to ~2 µM, and Ca^2+^ overspill from neighbouring Ca^2+^ channels, reduced peak Na^+^ currents, *I*_Na_, in Na_v_1.4-transfected HEK293 cells and skeletal muscle cell lines. These effects were abrogated by intracellular BAPTA^6^. Additionally, CaM expression in the transgenic cells negatively shifted the voltage-dependences of Na_v_1.4 inactivation. Both the reduction of peak *I*_Na_, and this negative shift in voltage dependence, were abolished by mutations in either the Ca^2+^-binding-EF hands on CaM or the Na_v_1.4 C-terminal IQ domain^2,6–8^. These results implicated CaM binding to the IQ domain in Na_v_1.4 modulation by [Ca^2+^]_i_.

Thirdly, increases in free intracellular concentrations of FK506 binding protein, [FKBP12]_i_ resulting from reductions in its affinity for RyR subunits associated with RyR opening^9,10^, positively shifted activation and inactivation voltage dependences in cardiomyocyte Na_v_1.5^11^, thought closely homologous to Na_v_1.4^4^. This likely involves an indirect effect; co-immunoprecipitation assays appear to exclude direct FKBP12–Na_v_1.5 interactions^11^.

However, fewer reports are available on such [Ca^2+^]_i_-dependent Na_v_1.4 modulation in intact *in situ* skeletal myocytes. Loose-patch clamp studies reported that carbonyl cyanide-3-chlorophenylhydrazone^12^ and 8-(4-chlorophenylthio)adenosine-3′, 5′-cyclic monophosphate challenge^13^ reduced *I*_Na_. These effects were reversed by the Ca^2+^ buffer BAPTA and RyR blocker dantrolene respectively. However, these agents affected [Ca^2+^]_i_ indirectly through modifying mitochondrial Ca^2+^ release or altering RyR activity through CAMKII-dependent pathways via Exchange protein directly activated by cAMP (Epac).

We here test the hypothesis that the *direct* RyR-SR Ca^2+^ channel modulator caffeine^14^ modifies *I*_Na_ in *intact* mammalian skeletal muscle fibres. We utilised experimental protocols previously shown to accomplish contrasting effects of 0.5 and >1.0 mM caffeine challenge on spectrofluometrically measured resting skeletal myocyte [Ca^2+^]_i_^15–17^. Thus, in isolated rabbit skeletal SR or murine skeletal muscle fibre preparations, caffeine (0.5 mM) caused *increased* [Ca^2+^]_i_ (to ~300 nM) from resting levels (typically ~106 ± 2 nM in rat gastrocnemius and soleus muscle fibres^18^). This was persistent over the subsequent 3–10 min intervals investigated^15,16^. In contrast, ≥1.0 mM caffeine induced early [Ca^2+^]_i_ peaks followed by *reductions* to persistently below-resting [Ca^2+^]_i_ within 80–90 sec^16,17^. The latter was attributed to a slow RyR inactivation reducing channel open probabilities taking place over seconds. This property was previously demonstrated in sheep and rabbit skeletal muscle RyRs reconstituted in lipid bilayers under levels of steady [Ca^2+^]_i_ that would initially produce RyR activation (~10–100 µM)^19^.

The present experiments correspondingly investigated effects of 0.5 mM and 2.0 mM caffeine on *I*_Na_ in intact murine skeletal muscle using the loose-patch clamp approach. This employed relatively low-resistance seals (<2 MΩ) with an undisrupted surface membrane thus leaving intracellular, particularly [Ca^2+^]_i_ homeostasis unperturbed. In contrast, conventional tight-patch seals often include the Ca^2+^-sequestrating ethylene glycol-bis(βaminoethyl ether)-N, N, N’, N’-tetraacetic acid (EGTA) in intracellular pipette solutions. In addition, the loose-patch pipettes could be re-used between patches, permitting sequential and multiple recordings from the same cell before and after pharmacological challenge. This permitted standardized comparisons between successive results with the same pipette^20^. Test results were compared with effects of control challenges using the known specific RyR blocker dantrolene^21^ to identify caffeine effects specifically attributable to its direct RyR action.

## Results

### Currents obtained using the double pulse protocol

**Figure 1:**
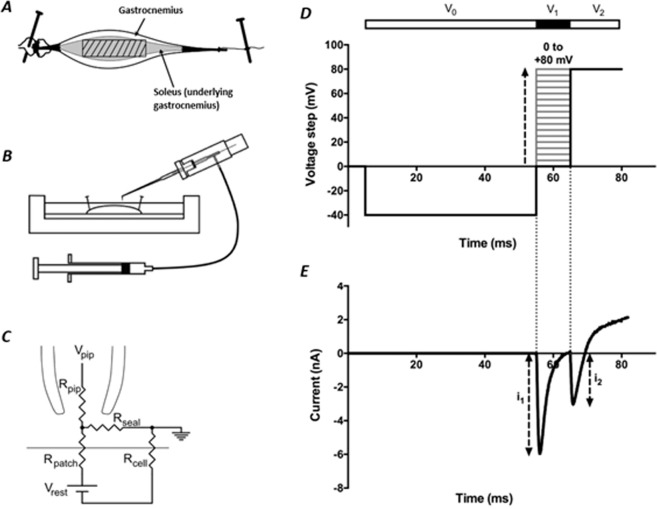
(**A**) Plan view of the muscle preparation after being pinned down at both ends in the bath. The soleus muscle (in grey) is overlaid by gastrocnemius. The shaded box in the centre represents the region of gastrocnemius fibres used for patch clamping. (**B**) Loose patch clamp experiment: Muscle preparation pinned down in KH solution. Pipette mounted at 45° to allow 90° contact of the bent tip with the fibre surface. The pipette is connected to a suction syringe^13^. (**C**) Equivalent circuit of loose patch clamp electrode on muscle membrane. Solution in the pipette clamped at *V*_pip_. The voltage error arising from the flow of currents through the patch resistance (*R*_patch_) and the cell resistance (*R*_cell_), along with the pipette resistance (*R*_pip_) corrected mainly using a bridge circuit in the loose patch clamp amplifier^13^. (**D**) Complete set of pulse protocols carried out on a patch being investigated. This double test pulse protocol first superimposed a −40 mV hyperpolarising pre-pulse, lasting 50 ms, on the resting potential, RMP. This was followed by a depolarising *V*_1_ pulse varying in amplitude to test voltages between RMP and (RMP + 80) mV in +5 mV increments. This was followed by a *V*_2_ pulse to a strongly depolarised potential of (RMP + 80) mV. (**E**) The corresponding current trace during a single sweep in the protocol under control conditions where *V*_1_ = (RMP + 35) mV, following P/4 protocol correction, giving peak currents *I*_1_ and *I*_2_.

The experiments employed a loose-patch clamp preparation and recording layout fully described in the Materials and Methods section (Fig. 1). Voltage-dependences of Na^+^ current, *I*_Na_, including its activation and inactivation, were examined using a double test pulse protocol (Fig. 1D). In each sweep of the full protocol, the membrane was first held at resting membrane potential (RMP) for 5 ms. A hyperpolarising pre-pulse, duration 50 ms, imposed at times 5–55 ms from the beginning of the sweep to voltage *V*_0_ = (RMP–40) mV, first removed any residual *I*_Na_ inactivation at the RMP. This standardized the proportion of activatable Na^+^ channels prior to application of the subsequent depolarising *V*_1_ pulse, itself of duration 10 ms, at time 55–65 ms.

At early intervals immediately following solution change, a simplified protocol examined *I*_Na_ in individual patches. This averaged results from 5 sweeps of a pulse protocol imposing a single voltage step to a single voltage *V*_1_ at (RMP + 80) mV between 55 and 80 ms. *I*_Na_ features at later time intervals following solution change were studied using the full procedure. This examined effects of a family of *V*_1_ amplitudes varied between sweeps in +5 mV increments through test voltages between RMP and (RMP + 80) mV. This elicited currents reflecting the voltage dependence of *I*_Na_ activation. A further *V*_2_ pulse was then made to a constant, depolarised voltage of (RMP + 80) mV. This would activate all the Na^+^ channels within the patch not inactivated by the preceding *V*_1_ pulse. This made it possible to explore the voltage dependence of Na^+^ current inactivation. For each value of *V*_1_, currents from 10 sweeps were averaged before examining the next value of *V*_1_. Each complete protocol applied to a single patch therefore involved collection of currents from 170 sweeps.

Figure 1E exemplifies currents obtained by a single sweep at a single voltage, *V*_1_ = (RMP + 35) mV following P/4 correction under control conditions. Downward, negative deflections denote inward while upward, positive deflections represent outward membrane currents. The *V*_0_ pulse typically yielded no currents. The *V*_1_ and associated *V*_2_ steps each elicited transient inward currents representing the opening and inactivation of Na^+^ channels. The magnitudes of their peaks, *i*_1_ and *i*_2_, were measured relative to the currents measured just before the respective *V*_1_ and *V*_2_ steps.

Each patch was only subjected to a single application of the complete set of pulse protocols. This made differences between results arising from prolonged changes in the patch such as membrane blebbing unlikely^22^. Furthermore, following every set of sweeps a further bracketing protocol was then imposed using a single *V*_1_ step to RMP. The resulting average *i*_2_ value from the succeeding *V*_2_ step was then compared with the *i*_2_ value obtained by the first 10 sweeps of the preceding test protocol, for which *V*_1_ was also RMP. Patch stability was confirmed, permitting further analysis, if the bracketing *i*_2_ value was within 10% of the test *i*_2_ value. Thus, only patches with a stable seal, *I*_Na_ generation, and activation and inactivation characteristics were selected for further analysis.

### Early effects of caffeine on Na^+^ currents

**Figure 2:**
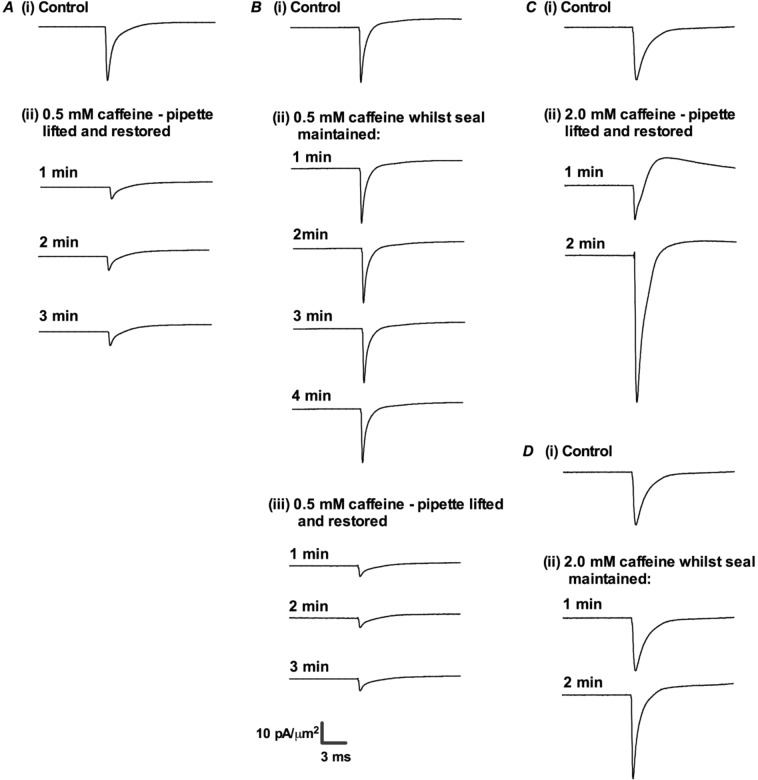
Results of simple pulse protocols to examine *I*_Na_ at early times following caffeine challenge using two experimental procedures. Following obtaining control records prior to caffeine challenge (**A**–**D**) panel (i), in (**A**), the patch electrode was withdrawn prior to introduction of 0.5 mM caffeine, and then restored prior to imposition of test steps (ii) in the following 1–3 min. In (**B**) the patch seal was maintained before, during and following introduction of 0.5 mM caffeine through imposition of the test steps (ii). The latter procedure was repeated following pipette withdrawal and restoration of the patch seal (iii). (**C**,**D**) adopted identical respective procedures as (**A**,**B**) using a 2 mM caffeine concentration.

The initial experiments explored for alterations in *I*_Na_ at the earliest times (1–4 min) following introduction of caffeine. The simplified protocol employing a single *V*_1_ test voltage of (RMP + 80) mV permitted prompt assessments of *I*_Na_ at times immediately following solution change. This made it additionally possible to explore the effects of using differing patch-clamping protocols either excluding or permitting caffeine access to membrane beneath the patch seal. All these manoeuvres first obtained control currents in the absence of caffeine (Fig. 2(A–D)(i)). The *I*_Na_ records showed increasing followed by decaying phases, conforming to previously established expectations of a voltage-dependent activation followed by more gradual inactivation of *I*_Na_. In a first procedure, the patch pipette was then withdrawn, 0.5 mM caffeine was introduced, and the patch seal re-established 30 sec later. This procedure permitted caffeine access to membrane both beyond and within the patch seal, prior to further applications of the pulse procedures (Fig. 2A(i)). The challenge with 0.5 caffeine then produced reductions in *I*_Na_ from the first minute of exposure (Fig. 2A(ii)).

A second procedure on a different patch employing a differing procedure yielded contrasting results. Following acquisition of control records (Fig. 2B(i)), 0.5 mM caffeine-containing extracellular solution was introduced whilst the pipette seal was maintained intact. Currents were similarly obtained at timed subsequent intervals. Here, the patch seal was accordingly continually maintained before, during and following introduction of caffeine-containing extracellular solution. This gave caffeine immediate access to membrane beyond but not within the patch seal. Challenge by 0.5 mM caffeine now produced little subsequent, early change in peak currents even with repeated *I*_Na_ determinations made at 1 min intervals between 1 to 4 minutes (Fig. 2B(ii)). In contrast, the effect of 0.5 mM caffeine in reducing *I*_Na_ could be finally restored in subsequent recordings made after lifting the patch pipette and restoring the patch seal 30 s later (Fig. 2B(iii)).

Separate experiments using 2.0 mM caffeine challenge gave concordant, complementary, results. Relative to the initial recordings in the absence of caffeine (Fig. 2C(i)), 2.0 mM caffeine initially produced a reduced *I*_Na_, but this was rapidly succeeded by increased *I*_Na_ if the latter test recordings were made after lifting then restoring the patch seal (Fig. 2C(ii)). These effects were attenuated when the 2.0 mM caffeine was added then test recordings made whilst contrastingly maintaining an intact patch seal (Fig. 2D(i,ii)).

These findings suggested that both 0.5 and 2 mM caffeine reduced *I*_Na_ within the first minute of exposure. This reduction persisted over 1, 2 and 3 min with 0.5 mM caffeine, but was succeeded by a marked increase in *I*_Na_ with 2 mM caffeine. However, such effects required a direct access of the caffeine to the area of membrane beneath the patch pipette in which *I*_Na_ was recorded. The subsequent explorations of caffeine effects on *I*_Na_ using the full pulse procedure at the later times accordingly challenged with caffeine prior to formation of the patch seal.

### Effects on I_Na_ magnitudes of 0.5 mM caffeine challenge

**Figure 3:**
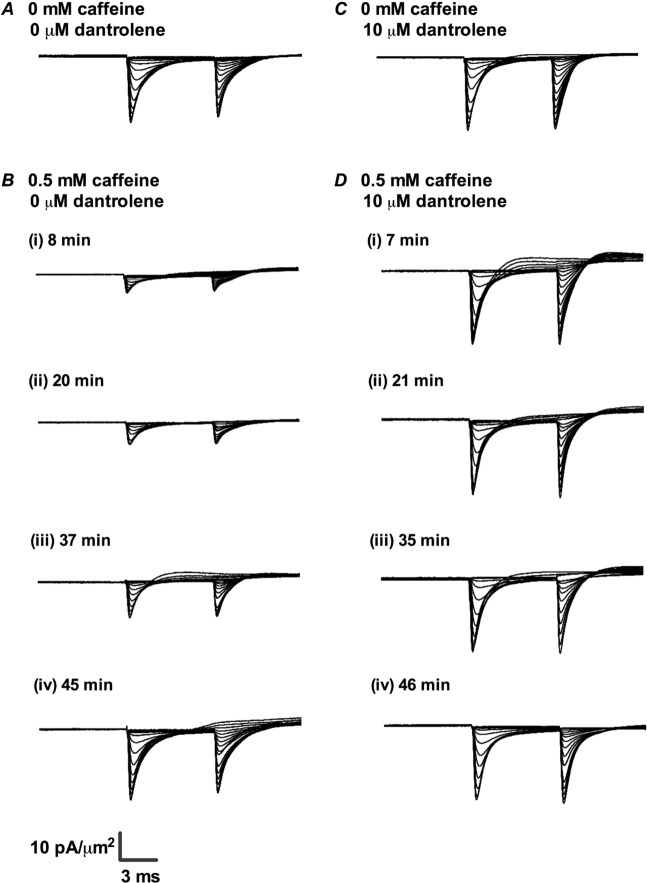
Families of normalized current traces obtained from the double pulse protocol in response to caffeine challenge at a concentration of 0.5 mM in the absence (**A**,**B**) or presence (**C**,**D**) of 10 μM dantrolene. (**A**,**C**) Control traces obtained in KH solution, prior to caffeine challenge in the absence (**A**) or presence of dantrolene (**C**). (**B**,**D**) Families of records obtained at successive (i) 7–8 min, (ii) 20–21 min, (iii) 35–37 min and (iv) 45–46 min intervals after addition of 0.5 mM caffeine-containing KH solution into the bath in the absence (**B**) or presence of dantrolene (**D**).

The *I*_Na_ recordings showed patterns that paralleled the previously reported contrasting [Ca^2+^]_i_ changes produced by 0.5 and 2.0 mM caffeine concentrations. Figure 3 illustrates current records from families of voltage steps at differing *V*_1_ between RMP and (RMP + 80) mV under control conditions (A) and at different times following introduction of 0.5 mM caffeine (B). Initial rates of rise of current increased non-linearly with degree of depolarization elicited by the *V*_1_ steps. So did the peak *I*_Na_ which then reached a maximum at *V*_1_ ≈ (RMP + 75) mV before decreasing with further depolarisation. The subsequent *V*_2_ steps yielded currents whose peak *I*_Na_ decreased with increasingly positive *V*_1_ consistent with prior voltage-dependent channel inactivation reducing the proportion of remaining activatable Na^+^ channels. The pulse protocols were repeated on different patches over successive time intervals following the introduction of caffeine. Figure 3B((i–iv)) illustrates typical families of traces obtained from 8, 20, 37 and 45 min following introduction of caffeine. Comparison of the successive sets of records suggested that peak *I*_Na_ initially showed marked, ~80%, reductions within the 10-minute interval following challenge (Fig. 3B(i–ii)). This was followed by a recovery which then returned *I*_Na_ to pre-treatment levels over a >40 min time-course (Fig. 3B(iii–iv)).

### Changes in the voltage dependence of I_Na_ resulting from 0.5 mM caffeine challenge

**Figure 4:**
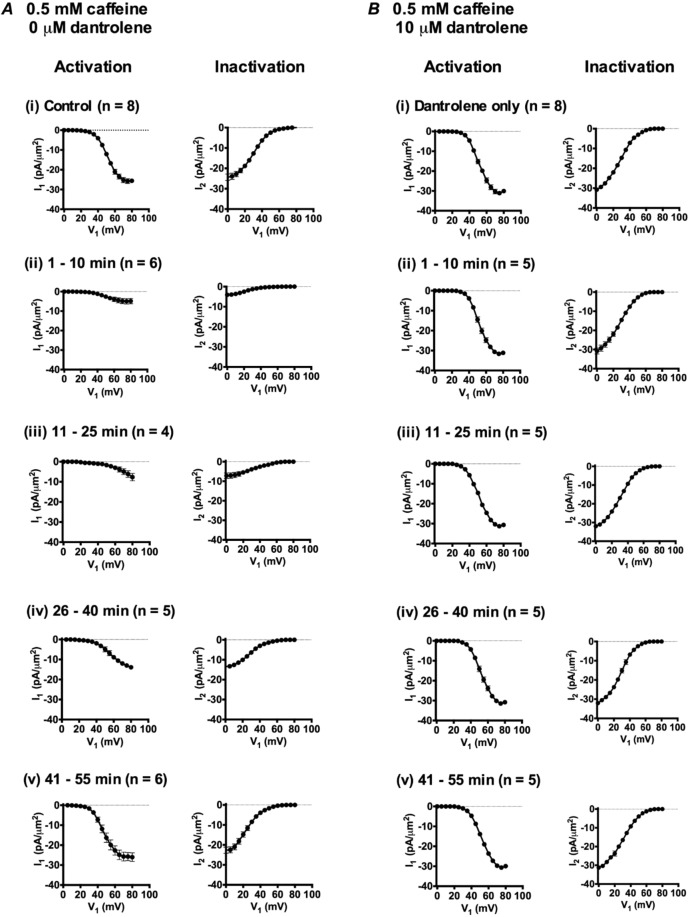
Activation (left panels) and inactivation *I*-*V* curves (right panels of each pair) (mean of *I*_1_ or *I*_2_ ± standard error of mean (SEM) against each *V*_1_ excursion) in response to caffeine challenge at a concentration of 0.5 mM in the absence (**A**) or the presence (**B**) of 10 μM dantrolene. Experiments first obtained families of *I*_Na_ records before addition of caffeine in (**A**(i)), (**B**(i)). Families of *I*_Na_ records then obtained in intervals (ii) 1–10 minutes, (iii) 11–25 minutes (iv) 26–40 minutes and (v) 41–55 min intervals after adding 0.5 mM caffeine.

**Figure 5:**
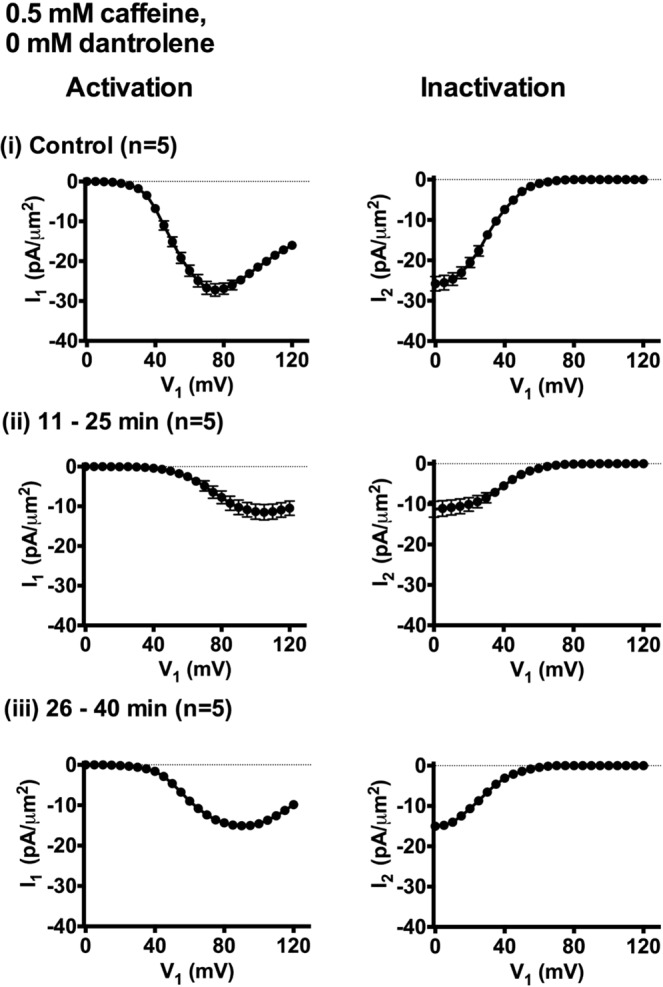
Activation and inactivation *I-V* curves (mean of *I*_1_ or *I*_2_ ±standard error of mean (SEM) against each *V*_1_ excursion) for the (i) control condition prior to addition of caffeine, and (ii), 11–25 minutes and (iii) 26–40 minutes after addition of 0.5 mM caffeine using an expanded range of *V*_1_ in the absence of dantrolene.

Findings of this kind yielded *I*_Na_ current-voltage activation (*I*-*V*) and inactivation curves plotting dependences of peak *I*_Na_ on *V*_1_ in response to the respective *V*_1_ and *V*_2_ voltage steps. These are plotted for families of records obtained at different intervals following onset of caffeine challenge (Fig. 4). These curves demonstrated that compared to pre-treatment controls (Fig. 4A(i)), *I*_Na_ was reduced at 1–10, 11–25 and 26–40 min (Fig. 4A(ii–iv) respectively). However *I*_Na_ had recovered by the final (41–55 min) time interval (Fig. 4A(iv)). The maximum values of peak *I*_Na_ could be determined within the voltage range examined in the *I-V* curves obtained in the controls at 1–10 and 41–55, but not 11–25 and 26–40 min. Accordingly, the same determinations were made under control conditions and at 11–25, and 26–40 minutes with a modified pulse protocol (Fig. 5) that explored an increased *V*_1_ range extending to (RMP + 120) mV and using a *V*_2_ step to (RMP + 100) mV. This encompassed the *V*_1_ at which maximum peak *I*_Na_ was observed, both under the control conditions (Fig. 5(i)) and at the selected times following addition of caffeine (Fig. 5(ii,iii)). Together the *I-V* plots suggested that the greatest peak *I*_Na_ occurred with depolarisations to (RMP + 105) mV at 11–25 min and at (RMP + 95) mV at 26–40 min respectively.

**Figure 6:**
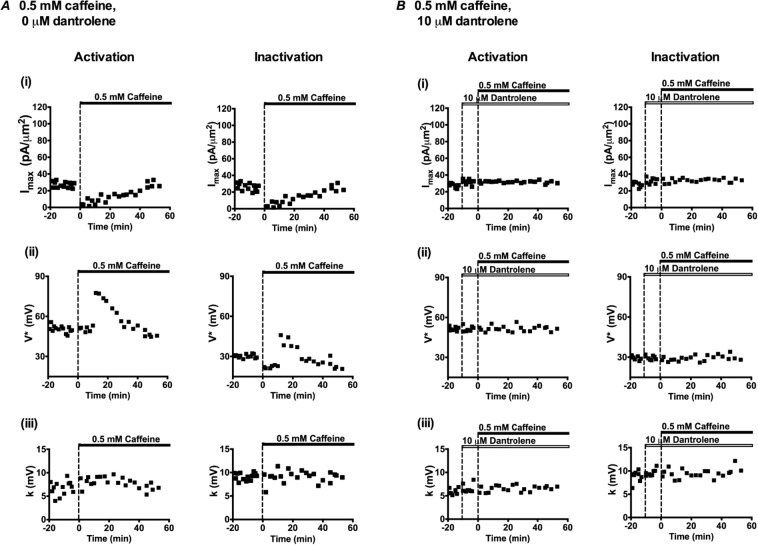
Alterations in (i) *I*_max_, (ii) V* and (iii) *k* derived from current-voltage (*I*-*V*) activation and inactivation curves followed from successive patches studied at different times before (t < 0 min) and following addition of 0.5 mM caffeine in the absence (**A**) and presence (**B**) of 10 μM dantrolene.

Figure 6A plots values of these parameters in successive patches with time following application of caffeine. Tables 1A and 2A list these parameters as means ± SEM from explorations grouped in successive time intervals as adopted in Fig. 3. This made it possible to test for statistically significant differences from the control findings obtained before addition of 0.5 mM caffeine. These demonstrated that within 1–10 min after adding caffeine, there was a sharp reduction in *I*_max_. In addition, there was a negative shift in the V* value describing *I*_Na_ inactivation. In contrast, V* values for *I*_Na_ activation and *k* values for both activation and inactivation then remained unchanged. There then followed marked positive shifts in the V* of both *I*_Na_ activation and *I*_Na_ inactivation. The final *I*_Na_ recovery to pretreatment values, was then accompanied by more gradual negative shifts in the voltage dependence of both *I*_Na_ activation and *I*_Na_ inactivation, with control *I*_max_, and V* values regained at 26–40 and 41–55 min respectively. These changes in *I*_max_ and V* contrasted with the constant *k* values through these manoeuvres. Furthermore, they were abolished, as described fully below, by dantrolene-mediated RyR block.

**Table 1:** Mean *I*_max_, V^*^ and *k* values of activation I-V curves at increasing time intervals following addition of 0.5 mM caffeine in the presence of (A) 0 μM dantrolene and (B) 10 μM dantrolene.

Time after adding caffeine (min)	*I*_max_, mean ± SEM (n- value), pA/µm^2^; *p-*value	V^*^, mean ± SEM (n -value), mV; *p-*value	*k*, mean ± SEM (n- value), mV; *p-*value
*(A) Experiments performed with 0.5 mM caffeine, 0 μM dantrolene*			
Control	27.15 ± 0.971 (n = 13)	50.47 ± 0.721 (n = 13)	6.720 ± 0.412 (n = 13)
1–10	5.14 ± 1.448 (n = 6)* P < 0.0001	50.75 ± 0.749 (n = 6) P = 0.8182	7.90 ± 0.455 (n = 6) P = 0.1015
11–25	11.65 ± 1.964 (n = 5)* P < 0.0001	73.16 ± 2.099 (n = 5)* P < 0.0001	9.02 ± 0.286 (n = 5) P = 0.0546
26–40	15.30 ± 0.875 (n = 5)* P < 0.0001	57.33 ± 2.115 (n = 5)* P = 0.0011	8.07 ± 0.274 (n = 5) P = 0.0709
41–55	26.52 ± 1.970 (n = 6) P = 0.7466	47.45 ± 1.372 (n = 6)* P = 0.0461	6.72 ± 0.346 (n = 6) P = 0.9974
*(B) Experiments performed with 0.5 mM caffeine, 10 μM dantrolene*			
Dantrolene alone	31.80 ± 1.086 (n = 8)	51.32 ± 0.703 (n = 8)	6.55 ± 0.330 (n = 8)
1–10	31.92 ± 0.516 (n = 5) P = 0.9322	51.70 ± 1.076 (n = 5) P = 0.7648	6.10 ± 0.297 (n = 5) P = 0.3714
11–25	31.77 ± 0.467 (n = 5) P = 0.9831	50.80 ± 0.720 (n = 5) P = 0.6365	6.86 ± 0.235 (n = 5) P = 0.5119
26–40	31.91 ± 0.448 (n = 5) P = 0.9401	52.08 ± 1.268 (n = 5) P = 0.5802	6.47 ± 0.359 (n = 5) P = 0.8808
41–55	31.27 ± 1.019 (n = 5) P = 0.7481	51.80 ± 0.797 (n = 5) P = 0.668	6.77 ± 0.112 (n = 5) P = 0.6148

P values indicate significance of differences compared to control values. *Denotes a significant difference compared to controls where P < 0.05.

**Table 2:** Mean *I*_max_, V^*^ and *k* values of inactivation I-V curves at increasing time intervals following addition of 0.5 mM caffeine in the presence of (A) 0 μM dantrolene and (B) 10 μM dantrolene.

Time after adding caffeine (min)	*I*_max_, mean ± SEM (n-value), pA/µm^2^; *p-*value	V^*^, mean ± SEM (n-value), mV; *p-*value	*k*, mean ± SEM (n-value), mV; *p-*value
*(A) Experiments performed with 0.5 mM caffeine, 0 μM dantrolene*			
Control	26.24 ± 1.245 (n = 13)	30.22 ± 0.364 (n = 13)	9.15 ± 0.217 (n = 13)
1–10	4.50 ± 1.202 (n = 6)* P < 0.0001	22.27 ± 0.399 (n = 6)* P < 0.0001	9.00 ± 0.733 (n = 6) P = 0.8105
11–25	11.33 ± 1.959 (n = 5)* P < 0.0001	40.68 ± 1.843 (n = 5)* P < 0.0001	9.28 ± 0.516 (n = 5) P = 0.7797
26–40	16.09 ± 0.896 (n = 5)* P = 0.0002	26.71 ± 0.730 (n = 5)* P = 0.0002	9.31 ± 0.563 (n = 5) P = 0.7361
41–55	24.90 ± 1.614 (n = 6) P = 0.5421	24.03 ± 1.526 (n = 6)* P < 0.0001	9.00 ± 0.344 (n = 6) P = 0.7255
*(B) Experiments performed with 0.5 mM caffeine, 10 μM dantrolene*			
Dantrolene alone	32.17 ± 1.225 (n = 8)	29.71 ± 0.555 (n = 8)	9.62 ± 0.258 (n = 8)
1–10	32.00 ± 1.535 (n = 5) P = 0.9305	27.97 ± 0.651 (n = 5) P = 0.071	9.77 ± 0.358 (n = 5) P = 0.7351
11–25	33.05 ± 0.913 (n = 5) P = 0.6227	30.38 ± 0.630 (n = 5) P = 0.4549	9.63 ± 0.286 (n = 5) P = 0.9975
26–40	32.55 ± 0.528 (n = 5) P = 0.8232	28.68 ± 1.154 (n = 5) P = 0.3855	8.48 ± 0.320 (n = 5) P = 0.187
41–55	32.52 ± 1.213 (n = 5) P = 0.8541	30.11 ± 1.056 (n = 5) P = 0.7191	10.12 ± 0.538 (n = 5) P = 0.3697

P values indicate significance of differences compared to control values. *Denotes a significant difference compared to controls where P < 0.05.

### Changes in magnitude and voltage dependence of I_Na_ resulting from 2 mM caffeine challenge

**Figure 7:**
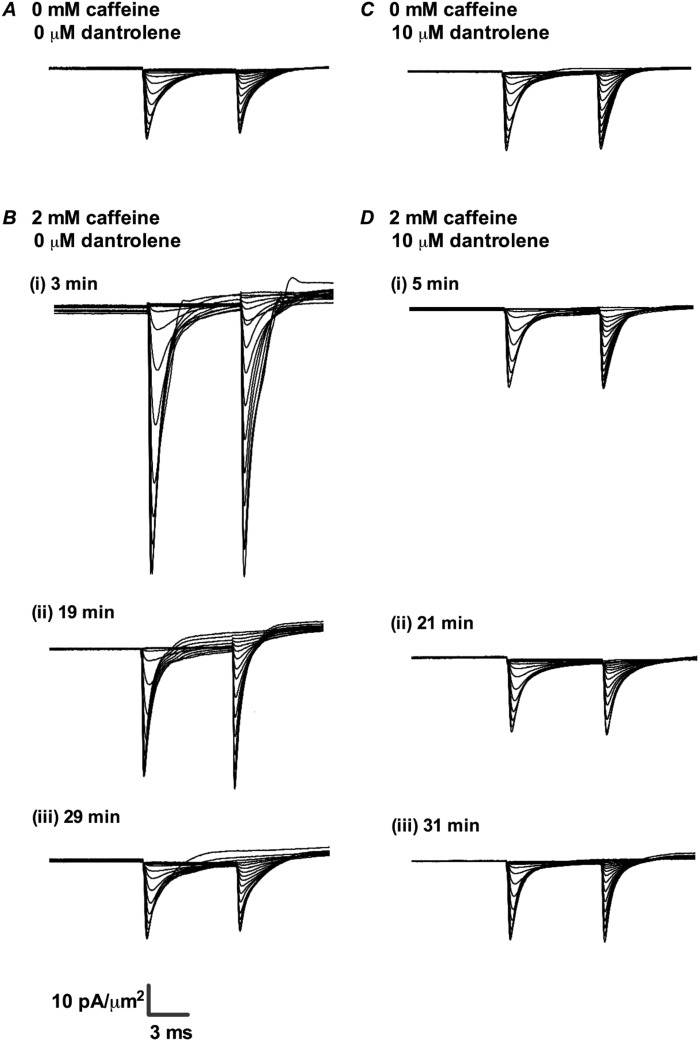
Families of normalized current traces obtained from the double pulse protocol in response to caffeine challenge at a concentration of 2 mM in the absence (**A**,**B**) or the presence (**C**,**D**) of 10 μM dantrolene. (**A**,**C**) Control traces obtained in KH solution, prior to caffeine challenge. (**B**,**D**) Families of records obtained at successive (i) 3 or 5 min, (ii) 19 or 21 min and (iii) 29 or 31 min after addition of 2 mM caffeine-containing KH solution into the bath in (**B**) the absence or (**D**) presence of dantrolene.

**Figure 8:**
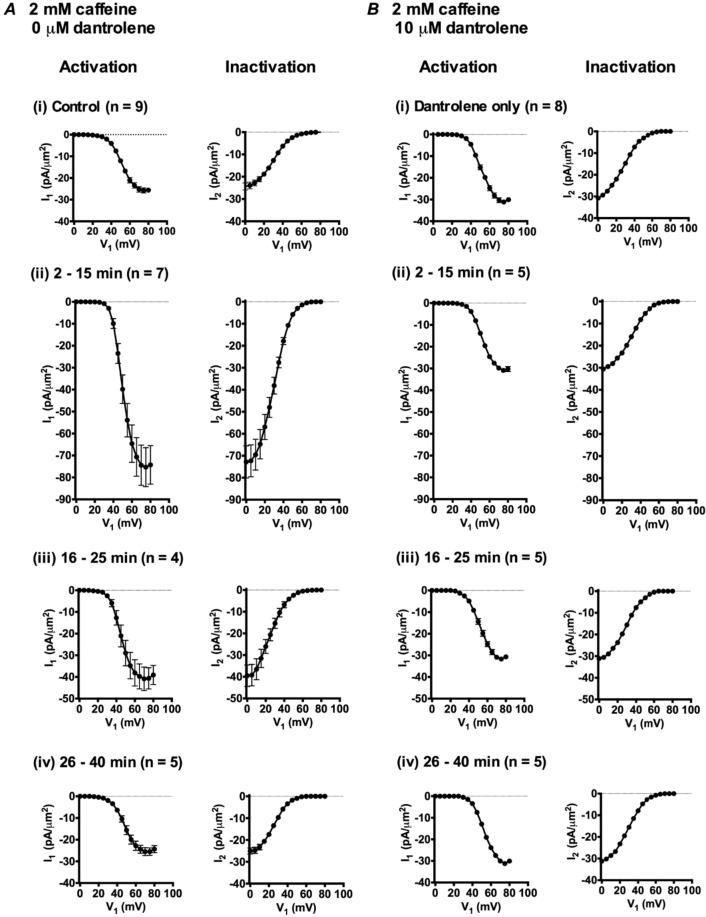
Activation (left panels) and inactivation *I*-*V* curves (right panels) (mean of *I*_1_ or *I*_2_ ± standard error of mean (SEM)) against each *V*_1_ excursion before (i) and following (ii) introduction of 2 mM caffeine in the absence (**A**) or the presence (**B**) of 10 μM dantrolene. Families of *I*_Na_ records were obtained 2–15 min (ii), 16–25 min (iii), and 26–40 min (iv) after adding 2 mM caffeine.

**Figure 9:**
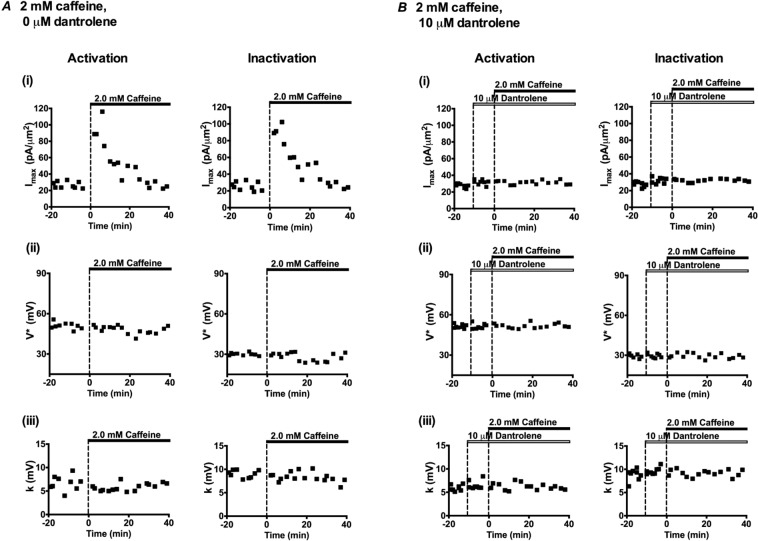
Alterations in (i) *I*_max_, (ii) V* and (iii) *k* derived from current-voltage (*I*-*V*) activation (left panels) and inactivation (right panels) followed from successive patches studied at different times before (t < 0 min) and following addition of 2 mM caffeine in the absence (**A**) and presence (**B**) of 10 μM dantrolene.

Caffeine challenge at a 2 mM concentration gave contrasting *I*_Na_ changes. Following the initially decreased peak *I*_Na_ within 2 min described above (Fig. 2C), the pattern assumed a transient increase in *I*_Na_ followed by its recovery to control levels. Figure 7 shows families of *I*_Na_ records obtained using the full pulse protocol as in Fig. 3 at the different times subsequent to 2.0 mM caffeine challenge. The current waveforms increased to a peak *I*_Na_ followed by an inactivating decline (Fig. 7A). However, the families of currents demonstrated markedly greater *I*_Na_ at 2–15 min following exposure to 2 mM caffeine, in contrast to the reduction in *I*_Na_ produced by 0.5 mM caffeine. *I*_Na_ then progressively declined to pre-treatment levels over the succeeding time intervals (Fig. 7B(i–iii)). Figure 8A plots the resulting *I-V* and inactivation curves obtained at different time intervals, from which it was possible to obtain *I*_max_, V* and *k* values for the *I-V* and inactivation curves. Figure 9 plots these values for successive patches with time following application of caffeine and Tables 3A and 4A summarise the statistical analysis of their means ± SEM from explorations in the successive time intervals, comparing these against the control pretreatment results.

**Table 3:** Mean *I*_max_, V^*^ and *k* values of activation *I*-*V* curves at particular time intervals after addition of 2 mM caffeine in the presence of (A) 0 μM dantrolene and (B) 10 μM dantrolene.

Time after adding caffeine (min)	*I*_max_, mean ± SEM (n-value), pA/µm^2^; *p-*value	V^*^, Mean ± SEM (n-value), mV; *p-*value	*k*, mean ± SEM (n-value), mV; *p-*value
*(A) Experiments performed with 2 mM caffeine, 0 μM dantrolene*			
Control	26.87 ± 1.346 (n = 9)	51.01 ± 0.839 (n = 9)	6.71 ± 0.518 (n = 9)
2–15	75.56 ± 9.026 (n = 7)* P < 0.0001	49.99 ± 0.568 (n = 7) P = 0.3577	5.35 ± 0.130 (n = 7) P = 0.0507
16–25	41.07 ± 4.714 (n = 4)* P = 0.0023	45.70 ± 1.711 (n = 4)* P = 0.009	5.85 ± 0.622 (n = 4) P = 0.3531
26–40	27.18 ± 1.844 (n = 5) P = 0.8952	47.37 ± 1.087 (n = 5)* P = 0.0223	6.49 ± 0.159 (n = 5) P = 0.7647
*(B) Experiments performed with 2 mM caffeine, 10 μM dantrolene*			
Dantrolene alone	31.80 ± 1.086 (n = 8)	51.32 ± 0.703 (n = 8)	6.55 ± 0.330 (n = 8)
1–15	31.14 ± 0.985 (n = 6) P = 0.6755	51.20 ± 0.643 (n = 6) P = 0.9072	6.31 ± 0.385 (n = 6) P = 0.6494
16–25	32.21 ± 1.186 (n = 4) P = 0.8186	51.94 ± 1.234 (n = 4) P = 0.6483	6.33 ± 0.374 (n = 4) P = 0.6954
26–40	31.17 ± 1.199 (n = 5) P = 0.7132	52.18 ± 0.650 (n = 5) P = 0.4217	6.02 ± 0.185 (n = 5) P = 0.2633

P values indicate significance of differences compared to control values. *Denotes a significant difference compared to controls where P < 0.05.

**Table 4:** Mean *I*_max_, V^*^ and *k* values of inactivation *I-V* curves at particular time intervals after addition of 2 mM caffeine in the presence of (A) 0 μM dantrolene and (B) 10 μM dantrolene.

Time after adding caffeine (min)	I_max_, mean ± SEM (n-value), pA/µm^2^; *p-*value	V^*^, Mean ± SEM (n-value), mV; *p-*value	*k*, mean ± SEM (n-value), mV; *p-*value
*(A) Experiments performed with 2 mM caffeine, 0 μM dantrolene*			
Control	25.79 ± 1.713 (n = 9)	30.03 ± 0.343 (n = 9)	9.00 ± 0.269 (n = 9)
2–15	75.24 ± 7.502 (n = 7)* P < 0.0001	30.22 ± 0.507 (n = 7) P = 0.7513	8.39 ± 0.282 (n = 7) P = 0.1422
16–25	43.01 ± 5.505 (n = 4)* P = 0.0022	24.45 ± 0.437 (n = 4)* P < 0.0001	9.06 ± 0.605 (n = 4) P = 0.9101
26–40	26.40 ± 1.579 (n = 5) P = 0.8169	27.40 ± 1.458 (n = 5)* P = 0.0411	7.62 ± 0.398 (n = 5) P = 0.0719
*(B) Experiments performed with 2 mM caffeine, 10 μM dantrolene*			
Dantrolene alone	32.17 ± 1.225 (n = 8)	29.71 ± 0.555 (n = 8)	9.62 ± 0.258 (n = 8)
1–15	31.43 ± 0.786 (n = 6) P = 0.6476	30.43 ± 0.755 (n = 6) P = 0.4431	9.05 ± 0.362 (n = 6) P = 0.2069
16–25	32.48 ± 1.056 (n = 4) P = 0.8756	28.44 ± 0.925 (n = 4) P = 0.238	9.20 ± 0.165 (n = 4) P = 0.3059
26–40	32.38 ± 0.595 (n = 5) P = 0.9029	29.06 ± 0.811 (n = 5) P = 0.509	9.19 ± 0.393 (n = 5) P = 0.358

P values indicate significance of differences compared to control values. *Denotes a significant difference compared to controls where P < 0.05.

The *I*_Na_ amplitudes, *I*_max_, increased threefold over the 2–15 min following addition of caffeine when compared to its control values (Compare Fig. 8A(i,ii)). *I*_max_ then recovered in subsequent intervals to regain pre-treatment values at 26–40 minutes (Fig. 8A(iii,iv)). There were delayed negative shifts in the V* of both *I*_Na_ activation and *I*_Na_ inactivation at 16–25 min followed by smaller positive shifts at 26–40 min ((Fig. 9A(ii)); Tables 3A and 4A). Again, in common with findings using 0.5 mM caffeine, values of *k* showed relatively little change through these protocols.

### Effect of dantrolene on the I_Na_ responses to 0.5 and 2.0 mM caffeine

The final sequence of experiments examined the effects of the specific RyR inhibitor dantrolene both by itself and on these divergent actions of 0.5 and 2.0 mM caffeine on *I*_Na_ using otherwise identical pulse protocols. Each experiment first obtained families of *I*_Na_ traces in the absence of all test agents in two successive patches studied within a first 10 min interval. The bath solution was then substituted for one containing 10 µM dantrolene and 2 further patches investigated within the next 10 minutes. The final *I*_Na_ records were then obtained after further replacing the bath solution with one containing both 0.5 or 2.0 mM caffeine and 10 µM dantrolene-containing solution. Patches were investigated using the same full pulse protocols as shown in Fig. 1.

Including dantrolene alone slightly increased peak *I*_Na_ (Figs. 3C and 7C). Analysis of the *I-V* relationships (Figs. 4B and 8B) accordingly demonstrated small but significant increases in activation and inactivation *I*_max_ compared to controls, but no effect on activation and inactivation V* and *k* values (Tables 5 and 6).

**Table 5:** Mean *I*_max_, V^*^ and *k* values of activation *I-V* curves before and after addition of 10 μM dantrolene.

Condition	*I*_max_, mean ± SEM (n-value), pA/µm^2^; *p-*value	V*, mean ± SEM (n-value), mV; *p-*value	*k*, mean ± SEM (n-value), mV; *p-*value
Control	27.67 ± 0.881 (n = 8)	51.39 ± 0.528 (n = 8)	5.92 ± 0.221 (n = 8)
Dantrolene	31.80 ± 1.086 (n = 8)* P = 0.0105	51.32 ± 0.703 (n = 8) P = 0.9346	6.55 ± 0.330 (n = 8) P = 0.1372

P values indicate significance of differences compared to control values. *Denotes a significant difference compared to controls where P < 0.05.

**Table 6:** Mean *I*_max_, V^*^ and *k* values of inactivation *I-V* curves before and after addition of 10 μM dantrolene.

Condition	I_max_, mean ± SEM (n-value), pA/µm^2^; *p-*value	V*, mean ± SEM (n-value), mV; *p-*value	*k*, mean ± SEM (n-value), mV; *p-*value
Control	27.66 ± 1.075 (n = 8)	29.64 ± 0.5001 (n = 8)	8.824 ± 0.4387 (n = 8)
Dantrolene	32.17 ± 1.225 (n = 8)* P = 0.0151	29.71 ± 0.5547 (n = 8) P = 0.9321	9.623 ± 0.2582 (n = 8) P = 0.1386

P values indicate significance of differences compared to control values. *Denotes a significant difference compared to controls where P < 0.05.

Simultaneous addition of dantrolene and 0.5 mM (Fig. 3C,D) or 2.0 mM caffeine (Fig. 7C,D) (Tables 1–4) resulted in currents whose magnitudes were similar to those obtained in the presence of dantrolene alone, at all the time intervals following the addition of caffeine. Dantrolene thus abrogated both the effects of 0.5 mM caffeine in producing transient decreases (Fig. 3D(i–iv)), and the effects of 2 mM caffeine in producing transient increases in *I*_Na_ (Fig. 7D(i–iii)). In parallel with the previous test experiments, *I*-*V* and inactivation relationships were plotted for fibres studied in the presence of dantrolene and either 0.5 mM (Figs. 4B) or 2.0 mM caffeine (Fig. 8B) through the same time intervals as those used after challenge with 0.5 mM or 2.0 mM caffeine alone, respectively.

Figures 6B and 9B plot the resulting *I*_max_, V* and *k* values for the *I-V* and inactivation curves for successive patches with time before, and following addition of either 0.5 (Figs. 6B) or 2 mM caffeine (Fig. 9B) in successive individual patches exposed to dantrolene. None of these parameters showed the contrasting pattern of time-dependent changes in activation and inactivation parameters shown with 0.5 and 2 mM caffeine challenge in the absence of dantrolene. Tables 1–4 summarise the statistical analysis of their means ± SEM from explorations in the successive time intervals comparing these (B) against the control pretreatment results (A). In both analyses the activation and inactivation *I*_max_, V^*^ and *k* values were respectively statistically indistinguishable from those obtained under dantrolene challenge alone when caffeine was absent. Again values of *k* remained constant through the experiments.

Neither challenge by dantrolene, nor that by caffeine, whether by itself or in combination with dantrolene, noticeably affected *I*_Na_ kinetics. When quantified by times to peak current at voltages yielding maximum *I*_Na_ in the *I-V* curves, control experimental groups subsequently used to test effects of dantrolene alone, or 0.5 or 2 mM caffeine in the absence of or following further dantrolene challenge, gave times of 0.49 ± 0.010, 0.48 ± 0.0093 and 0.50 ± 0.012 ms respectively. Dunnett multiple comparison tests demonstrated that none of the subsequent interventions produced significantly alterations for all the time intervals assessed in Tables 1–4 (all P ≫ 0.05).

These control experiments thus demonstrate that (a) the RyR inhibitor dantrolene abrogated the contrasting actions of either 0.5 mM or 2.0 mM caffeine in transiently decreasing or increasing *I*_Na_ with accompanying changes in V* to previously established altered RyR-mediated changes in [Ca^2+^]. (b) Their demonstration of an increased *I*_Na_ in the presence of dantrolene alone complements the demonstrations of increased *I*_Na_ produced by 2 mM caffeine in suggesting some background inhibition of *I*_Na_ at even background resting [Ca^2+^]_i_.

## Discussion

The present experiments explored for the existence of physiologically effective feedback loops between ryanodine receptor (RyR) activation by tubular depolarisation and Na_v_1.4 channel function that would thereby modify surface membrane excitability in skeletal muscle *in vivo*. This mechanism would complement feedforward, excitation-contraction-coupling, processes by which Nav1.4-mediated surface membrane depolarisation triggers RyR-mediated release of intracellularly stored sarcoplasmic reticular (SR) Ca^2+^. Feedback alterations in Na_v_1.4 availability and/or voltage sensitivity are potentially physiologically important particularly under conditions of intense and prolonged muscle activation including tetanic stimulation and metabolic exhaustion. The latter changes could compromise SR Ca^2+^-ATPase activity through ATP depletion, thereby elevating [Ca^2+^]_i_. Any consequent effects of Ca^2+^ on Nav1.4 function would complement its other actions promoting slow RyR inactivation^19^ and increasing sarcolemmal Ca^2+^-activated large conductance K^+^ channel (K_Ca_1.1) conductance in downregulating excitation-contraction coupling^23^. Such feedback mechanisms would also bear on clinical conditions affecting skeletal muscle excitability. K^+^-aggravated myotonia is associated with slowed *I*_Na_ kinetics and impaired *I*_Na_ inactivation that accompany Nav1.4 mutations in its EF-hand-like domain^24^. Other genetic, K^+^ and cold-aggravated myotonias are associated with reduced Ca^2+^-dependent Na^+^ channel inhibition similarly attributed to Nav1.4 mutations^6^. Finally, the weakness observed in dystrophic muscle is associated with elevated [Ca^2+^]_i_^25^.

The experiments here examined effects of direct pharmacological manipulation of RyR-mediated SR Ca^2+^ release by the RyR agonist caffeine^14^ on both Na_v_1.4 channel availability and its voltage-dependent activation and inactivation properties in intact skeletal myocytes. They utilised previously established contrasting effects of different applied caffeine concentrations on spectrofluometrically measured resting [Ca^2+^]_i_^15,16^. Low concentrations of caffeine (0.5 mM) then *increased* [Ca^2+^]_i_ persistently over the subsequent 3–10 min recording intervals in isolated rabbit skeletal SR or murine skeletal muscle fibre preparations^15^. In contrast, ≥1.0 mM caffeine induced early [Ca^2+^]_i_ peaks rapidly followed by [Ca^2+^]_i_ *decreases* below even resting-levels within 80–90 sec^16,17^.

Here, Na^+^ currents, *I*_Na_ were measured in intact mammalian gastrocnemius skeletal muscle using the loose patch clamp technique. This avoided membrane disruption and perturbations of [Ca^2+^]_i_ homeostasis inherent in other experimental measurement procedures involving conventional cell-attached patch clamp studies, which additionally often include the Ca^2+^-sequestrating EGTA in the pipette solutions. Furthermore, the latter can require study of isolated or cultured cells with enzymatically treated membranes as opposed to the intact *in situ* skeletal myocytes examined here. The loose-patch configuration also permitted sequential and multiple recordings with the same standardized pipette in successive patches before and after pharmacological challenge as required in the present experiments^26^. The features of the resulting *I*_Na_ concurred with those obtained on earlier occasions^26^ and with other voltage clamp methods applied to mammalian skeletal muscle^27^. The magnitude, voltage-dependence and quantified activation (current-voltage, *I*-*V*) and inactivation properties of *I*_Na_ were compared at different times following introducing caffeine. Further experiments blocking these effects with further applications of the specific RyR inhibitor dantrolene attributed the observed changes specifically to altered RyR-mediated SR Ca^2+^ release.

The observed contrasting alterations in *I*_Na_ closely paralleled the previously reported [Ca^2+^]_i_ changes following caffeine challenge. Thus, *I*_Na_ was reduced early (1–2 min) following either 0.5 or 2 mM caffeine challenge. This effect was abrogated by reducing access of caffeine to tubular and therefore tubular-sarcoplasmic reticular triad regions in which RyRs are concentrated, in regions within the patch from which the recordings were obtained. The latter was accomplished by establishing patch seals before rather than after introduction of caffeine to the extracellular space. This would alter direct access of caffeine over a surface membrane area close to the 30 μm^2^ cross sectional area of the electrode tip in contact with the membrane within which *I*_Na_ was recorded.

Subsequent recordings following the 0.5 and 2 mM caffeine challenge obtained by identical pulse protocols respectively demonstrated contrasting increases and decreases in peak *I*_Na_ followed by their recovery to pretreatment levels. These findings corroborate and extend previous independent reports that *I*_Na_ was reduced by increased RyR activity following 8-CPT induced Epac pathway activation^13^. The *I*_Na_ changes were quantified by systematically exploring a consistent range of *V*_1_ test voltages thereby deriving families of *I*_Na_ records. These in turn yielded activation, *I-V*, and inactivation curves giving *I*_max_, V* and *k* values. These respectively quantified total available *I*_Na_ and the position and steepness of their voltage dependences. Sustained reductions in *I*_max_ induced by 0.5 mM caffeine paralleled the sustained [Ca^2+^]_i_ increases previously reported following caffeine mediated activation of RyR-mediated SR Ca^2+^ release^15^. The increased *I*_Na_ observed in the presence of 2 mM caffeine similarly paralleled reported contrastingly decreased [Ca^2+^]_i_ attributed to slow RyR inactivation^16,17^. The latter findings further suggested significant inhibition of *I*_Na_ by even normal background [Ca^2+^]_i_.

These alterations in total available *I*_Na_ were accompanied by time-dependent changes in properties of the *I-V* and inactivation curves. With 0.5 mM caffeine challenge, the initial decreases in *I*_Na_ were accompanied by negative shifts in inactivation V* over 1–10 min. This was followed, over 11–25 min, by positive shifts in *both I*_Na_ activation and *I*_Na_ inactivation V*. The final *I*_Na_ recovery to pretreatment values between 26–55 min was accompanied by opposite, negative, shifts in activation and inactivation V*. With 2 mM caffeine challenge, the contrasting, declining phases of the transient *I*_Na_ increases accompanied delayed transient negative shifts in both activation and inactivation V*. In contrast, values of *k* of either activation or inactivation little changed throughout these protocols.

Finally, the effects of the specific RyR inhibitor dantrolene were investigated both by itself and in combination with identical caffeine concentrations and pulse protocols. Dantrolene specifically stabilises RyR closed states by selectively decreasing Ca^2+^ affinities of RyR activation sites^21^. Previous reports had demonstrated that it inhibited caffeine-induced increments in mammalian SR Ca^2+^ permeability^28^ and caffeine-induced contractures in murine diaphragm muscle^29^.

In the present experiments dantrolene abrogated all the effects of either 0.5 or 2 mM caffeine, whether on *I*_max_, or activation or inactivation *I*_Na_ or V*. In contrast, dantrolene by itself produced only small increases in *I*_max_ without affecting activation and inactivation, V* and *k* values. Together with the increased *I*_Na_ produced by 2 mM caffeine this latter finding suggests that [Ca^2+^]_i_ exerts significantly inhibits *I*_Na_ even at background RyR activity. These explorations using dantrolene thus trace all the present actions of caffeine on *I*_Na_ specifically to its effects on RyR-mediated SR Ca^2+^ release.

**Figure 10:**
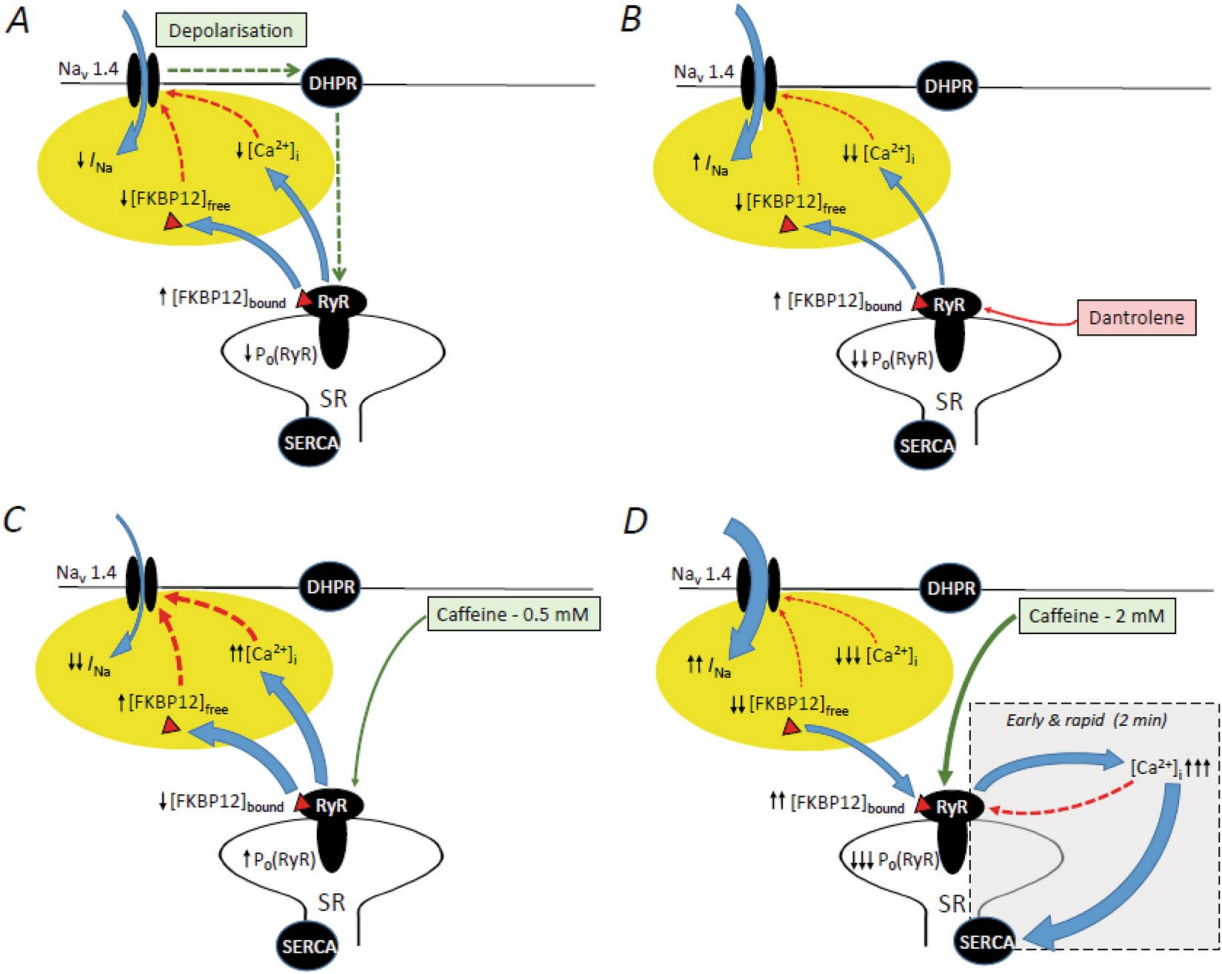
Correlation between observed ryanodine receptor (RyR) agonist and antagonist actions and (**A**) Feedforward Nav1.5 mediated depolarisation triggering DHPR-RyR coupling causing SR Ca^2+^ release that is (**B**) inhibited by RyR-antagonist dantrolene, and previous studies reporting: (**C**,**D**) Challenge by (**C**) 0.5 mM caffeine increasing RyR-mediated Ca^2+^ release and FKBP12 dissociation reducing Nav1.4 availability thereby modifying Nav1.4 properties and (**D**) 2 mM caffeine, where Ca^2+^ release causes sustained RyR inactivation with net SR Ca^2+^ uptake, reduced FKBP12 dissociation, and contrasting Nav1.4 effects. Blue arrows: fluxes; green and red dotted arrows: excitatory and inhibitory actions.

These findings together demonstrate and characterise RyR-Nav1.4 feedback phenomena complementing their established feedforward interactions underlying excitation contraction coupling (Fig. 10A). The observed contrasting effects of caffeine challenge on *I*_max_ and V* combined with previously reported [Ca^2+^]_i_ changes under similar experimental conditions^15–17^ were incompatible with straightforward combined negative shifts in Na^+^ channel activation and inactivation V* resulting from simple screening effects arising from an increased [Ca^2+^]_i_^30,31^. However, they directly correlate with previous *in vitro* biochemical and biophysical reports on skeletal muscle Na_v_1.4 and RyR. They establish for these a physiological context, possibly occurring within a common microdomain, in working muscle, as opposed to *in vitro* systems for the first time.

First, the early *I*_Na_ reductions with 0.5 and 2 mM caffeine, persistent with 0.5 mM caffeine, were consistent with the reported effects of either direct Ca^2+^-binding to a Na_v_1.4 EF hand site or indirect Ca^2+^ binding to Na_v_1.4 via its binding to CaM in reducing *I*_Na_ (Fig. 10C)_._ Secondly, the accompanying early negative V* shifts 1–10 min following 0.5 mM caffeine challenge paralleled reported negative shifts in voltage-dependent inactivation produced by the indirect Ca^2+^ binding process^2,6–8^. This was selective for Nav1.4 as opposed to Nav1.5, reflecting Nav1.4 lacking the CaM N-lobe binding site present in Nav1.5^1^. Thirdly, the subsequent (11–25 min) positive shifts in *I*_Na_ activation and inactivation V* directly paralleled reported reductions in FKPB12 binding affinity for RyR following RyR activation. An increased free [FKBP12]_i_ would then accompany the resulting SR Ca^2+^ release elevating [Ca^2+^]_i_. in intact fibres^9,10^; FKBP12 is known to positively shift V* activation and inactivation in Na_v_1.5^11^, thought closely homologous to Na_v_1.4^4^. This action is probably indirect and delayed: co-immunoprecipitation assays appeared to exclude direct FKBP12–Na_v_1.5 interactions^11^. Fourthly, negative shifts in *both I*_Na_ activation and inactivation V* values eventually accompanied the reductions in *I*_Na_ and recoveries from *I*_Na_ elevation, 26–55 or 16–40 min following 0.5 and 2.0 mM caffeine challenge respectively. These were compatible with a subsequent, previously reported, slow RyR inactivation^19^. This would reduce RyR open channel probabilities and Ca^2+^ release whilst permitting continued SR Ca^2+^ re-uptake and possible surface membrane Ca^2+^ expulsion by Na^+^/Ca^2+^ exchange^32,33^. These would reverse the increases in [Ca^2+^]_i_ and [FKBP12]_i_^19^ (Fig. 10D). Finally, the effects of the RyR inhibitor dantrolene (Fig. 10B) abrogating all these contrasting reductions and increases in *I*_Na_ associated with different caffeine concentrations through the identical pulse protocols associated all these findings with feedback effects arising specifically from the RyR.

## Materials and Methods

Experiments were performed under the Animals (Scientific Procedures) Act 1986 Amendment Regulations 2012 following ethical review by the University of Cambridge Animal Welfare and Ethical Review Body (AWERB). C57BL6 WT mice aged 3–6 months were used in all experiments. Mice were housed at a stable temperature of 21 °C in a licensed facility, fed sterile rodent chow (RM3 Maintenance Diet; SDS, Witham, United Kingdom) and subjected to 12-hour light/dark cycles with free access to water, bedding and environmental stimuli. Krebs–Henseleit (KH) solution (mmol/L: NaCl, 130; KCl, 4.0; HEPES, 1.2; MgCl_2_, 1.0; CaCl_2_, 1.8; glucose, 10; and Na-pyruvate, 2.0; pH adjusted to 7.4) was used in all experiments. Chemical agents were purchased from Sigma-Aldrich (Poole, UK) unless otherwise stated. The following variants of the basic KH solution containing caffeine and/or dantrolene including 0.1% dimethyl sulfoxide vehicle were prepared and used in experiments: (1) KH + 0.5 mM caffeine, (2) KH + 2.0 mM caffeine, (3) KH + 10 µM dantrolene, (4) KH + 10 µM dantrolene + 0.5 mM caffeine, and (5) KH + 10 µM dantrolene + 2.0 mM caffeine. Solutions were filtered to remove particles greater than 10 μm in diameter with standard filtration paper (Millipore, Bedford, MA, USA).

Mice were killed by cervical dislocation by Home Office-licensed personnel in accordance with Schedule 1 of The Animals (Scientific Procedures) Act 1986. The gastrocnemius and soleus muscles were then immediately isolated, with the distal (Achilles) tendon secured by a knot to a ligature and the proximal tendon still attached to its bony origin. Overlying connective tissue was excised to allow access to the myocyte surface. The isolated muscles were then immediately placed in a Sylgard-bottomed experimental bath containing KH solution with the soleus muscle facing down maximizing electrode access to gastrocnemius fibres. Experiments were carried out at stable temperatures around 20 °C. The muscle was secured in a taut and fixed position by pinning down the bony origin and the ligature with A1 insect pins (Fig. 1A).

Loose patch clamp pipettes were pulled from borosilicate glass capillaries (GC150‐10; Harvard Apparatus, Cambridge, UK) using a Brown–Flaming microelectrode puller (Model P‐97; Sutter Instrument Co. Novato, CA). They were mounted under a microscope with ×250 magnification with a calibrated eyepiece graticule, and a small indentation made on one side of the pipette using a diamond knife. A transverse force was then applied distal to the indentation, giving a fracture perpendicular to the long axis of the pipette. Squarely-broken tips were then fire‐polished under visual guidance at ×400 magnification using an electrically-heated nichrome filament to smooth the edges of the tip. The internal diameters of the pipettes were measured under ×1000 magnification. Pipettes of internal tip diameters between 28–38 µm were used in the experiments. Such loose patch recordings thus provide a complete representation of the properties of both surface and tubular membrane directly beneath these seals. Finally, the pipettes were bent at about 1 mm from the tip to make a 45° angle with the pipette’s long axis. This allowed the tips to contact the muscle surface at 90° when mounted at an angle of 45° below the horizontal on the recording amplifier head stage (Fig. 1B). The pipettes were mounted onto a pipette holder with an Ag/AgCl electrode held within the inside of the pipette. The distal half of the pipette was filled with solution from the bath, using suction provided by a syringe via an air-filled connection to the pipette holder. The bath was actively grounded at reference potential, using an Ag/AgCl electrode. These Ag/AgCl electrodes maintained electrical connection between the organ bath and pipette with the electronic circuit.

The pipette was lowered onto the gastrocnemius muscle membrane surface and gentle suction applied to allow seal formation around the resulting membrane patch. The central regions of the muscle (crosshatched region in scheme shown in Fig. 1A) were targeted for each experiment as this region was the most horizontal, permitting optimal (~90°) contact between pipette tip and muscle. The loose patch clamp configuration clamps the voltage on the extracellular side of the membrane within the pipette seal. Therefore, positive and negative potentials applied within the pipette respectively hyperpolarise and depolarise the membrane potential of the patch relative to the cell resting membrane potential (RMP). In this report, the signs of the voltage steps are inverted and membrane potentials expressed relative to RMP, a convention adopted by previous papers using this technique^12,13,20,26^. The Ag/AgCl electrode within the pipette both measured the intra-pipette potential relative to the ground reference potential of the bath and passed the clamp current.

Figure 1C shows the equivalent circuit of a typical patch. The loose patch technique involves a relatively low seal resistance (typically < 2 MΩ). This ohmic leakage and series resistance error [(seal resistance + patch resistance) and pipette resistance], along with additional pipette capacitance currents were largely corrected by a bridge circuit within the loose patch clamp amplifier^26^. The remaining linear leakage and capacitive currents were corrected using a P/4 protocol in which the test steps were followed by four control voltage steps a quarter the amplitude and of opposite signs to the test step. These were of a sign and/or amplitude that did not activate the voltage conductances, hence only eliciting leak currents. These responses were measured, summed and subtracted from the current values obtained from the original test step within the loose patch clamp^26^.

An IBM-compatible computer connected to the loose patch clamp amplifier, which was connected to the headstage that held the recording pipette was used to deliver voltage clamp steps relative to the RMP. Study of each patch commenced with strongly depolarising +80 mV, 15 ms pulses in order to confirm the presence of ion channels. Only patches showing clear, resolvable inward currents with kinetics characteristic of voltage-gated Na^+^ channels were investigated with the full set of pulse protocols. The patch clamp currents were sampled by the computer at a 50 kHz digital sampling rate and filtered with a DC-10 kHz bandwidth, using a 10 kHz Bessel low pass filter.

Experiments were initially performed with the muscle preparation immersed in control KH solution. Around 2–3 different patches were investigated using the complete set of pulses within 20 minutes after transferring the muscle preparation into the bath. Only muscles that gave stable control results were further studied in the presence of pharmacological challenge. For each pharmacological condition, data from patches collected from at least 3 muscle preparations were used. To achieve conditions comparable to previous studies on [Ca^2+^]_i_, the bathing solution was then rapidly replaced with KH solution containing either 0.5 mM and 2.0 mM caffeine. The resulting effects on Na^+^ current amplitude, and activation and inactivation characteristics were then examined at increasing times from a sequence of different patches following onset of caffeine challenge. Averaged currents from the 10 sweeps obtained for each pulse voltage within the experimental protocol were then normalised to the pipette tip cross-sectional area to give current densities, using the formula relating current density (in pA/µm^2^), measured current (in nA) and pipette diameter (in µm):

$${\rm{Current}}\,{\rm{density}}({\rm{pA}}/{\mu {\rm{m}}}^{2})=\{{\rm{measured}}\,{\rm{current}}({\rm{nA}})\times 4000\}/\{\pi \times {[{\rm{pipette}}{\rm{diameter}}(\mu {\rm{m}})]}^{2}\}$$

The resulting peak *I*_1_ and *I*_2_ current densities obtained from the *V*_1_ and *V*_2_ steps in the double pulse protocols generated activation and inactivation current-voltage (*I*-*V*) curves, respectively, for each patch. The rising phase of the activation *I-V* curve for each patch was fitted to a two-state Boltzmann function relating the peak current density for any *V*_1_ excursion (*I*_1_) to its maximum value (*I*_max_), the voltage at half maximal peak current (V*), the excursion corresponding to the *V*_1_ step (*V*) and the steepness factor (*k*): *I*_1_ = *I*_max_*/*[1 + exp{(V* − *V*)/*k*}]). The inactivation *I*-*V* curves were fitted to a similar Boltzmann function relating the peak current density *I*_2_ for the *V*_2_ step following any given *V*_1_ excursion to its maximum value (*I*_max_), the voltage at half maximal current (V^*^), the excursion of the *V*_1_ step (*V*) and a steepness factor (*k*): *I*_2_ = *I*_max_(1 − 1*/*[1 + exp{(V* − *V*)/*k*}]).

Successive families of ionic currents revealed alterations in current characteristics with time following caffeine challenge. These are represented by displaying currents, current-voltage curves and computed values of *I*_max_, V^*^ and *k* as obtained from each patch averaged to give means ± SEM for currents obtained within successive time intervals following introduction of pharmacological agents. Curve fitting procedures of the *I-V* curves used fitting algorithms in the open source programme SciDAVis (Version 1.25-mac). Derived parameters obtained under different conditions were tested for significant differences to a P < 0.05 significance level by ANOVA with a post-hoc Dunnett’s test.

### Ethical approval

This research has been regulated under the Animals (Scientific Procedures) Act 1986 Amendment Regulations 2012 following ethical review and approval by the University of Cambridge Animal Welfare and Ethical Review Body (AWERB). All procedures were completed by Home Office-licensed personnel and fell within the scope of Schedule 1 of the UK Animals (Scientific Procedures) Act (1986).

## Data Availability

The datasets generated during and/or analysed during the current study are available from the corresponding author on reasonable request.
